# 2,2,2-Trichloro-*N*-(3,4-dimethyl­phen­yl)acetamide

**DOI:** 10.1107/S1600536809013075

**Published:** 2009-04-18

**Authors:** B. Thimme Gowda, Sabine Foro, Hiromitsu Terao, Hartmut Fuess

**Affiliations:** a Department of Chemistry, Mangalore University, Mangalagangotri 574 199, Mangalore, India; b Institute of Materials Science, Darmstadt University of Technology, Petersenstrasse 23, D-64287 Darmstadt, Germany; c Faculty of Integrated Arts and Sciences, Tokushima University, Minamijosanjima-cho, Tokushima 770-8502, Japan

## Abstract

The conformation of the N—H bond in the title compound, C_10_H_10_Cl_3_NO, is *anti* to the C=O bond. The amide H atom exhibits both intra­molecular N—H⋯Cl and inter­molecular N—H⋯O hydrogen bonding. The latter inter­actions link the mol­ecules into infinite chains.

## Related literature

For the preparation of the title compound, see: Shilpa & Gowda (2007 ▶). For related structures, see: Gowda *et al.* (2007 ▶, 2008 ▶, 2009 ▶)
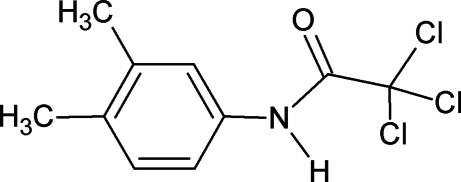


## Experimental

### 

#### Crystal data

C_10_H_10_Cl_3_NO
*M* *_r_* = 266.54
Monoclinic, 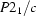

*a* = 5.9003 (8) Å
*b* = 20.843 (2) Å
*c* = 9.996 (1) Å
β = 105.53 (1)°
*V* = 1184.4 (2) Å^3^
*Z* = 4
Mo *K*α radiation
μ = 0.75 mm^−1^
*T* = 299 K
0.46 × 0.40 × 0.30 mm

#### Data collection

Oxford Diffraction Xcalibur diffractometer with a Sapphire CCD detector
Absorption correction: multi-scan (*CrysAlis RED*; Oxford Diffraction, 2007 ▶) *T*_min_ = 0.726, *T*_max_ = 0.807
9395 measured reflections
2407 independent reflections
1952 reflections with *I* > 2σ(*I*)
*R*_int_ = 0.015

#### Refinement

*R*[*F* ^2^ > 2σ(*F* ^2^)] = 0.057
*wR*(*F* ^2^) = 0.151
*S* = 1.08
2407 reflections
138 parameters
H-atom parameters constrained
Δρ_max_ = 0.96 e Å^−3^
Δρ_min_ = −0.88 e Å^−3^

### 

Data collection: *CrysAlis CCD* (Oxford Diffraction, 2004 ▶); cell refinement: *CrysAlis RED* (Oxford Diffraction, 2007 ▶); data reduction: *CrysAlis RED*; program(s) used to solve structure: *SHELXS97* (Sheldrick, 2008 ▶); program(s) used to refine structure: *SHELXL97* (Sheldrick, 2008 ▶); molecular graphics: *PLATON* (Spek, 2009 ▶); software used to prepare material for publication: *SHELXL97*.

## Supplementary Material

Crystal structure: contains datablocks I, global. DOI: 10.1107/S1600536809013075/bt2926sup1.cif

Structure factors: contains datablocks I. DOI: 10.1107/S1600536809013075/bt2926Isup2.hkl

Additional supplementary materials:  crystallographic information; 3D view; checkCIF report

## Figures and Tables

**Table 1:** Hydrogen-bond geometry (Å, °)

*D*—H⋯*A*	*D*—H	H⋯*A*	*D*⋯*A*	*D*—H⋯*A*
N1—H1*N*⋯O1^i^	0.86	2.14	2.917 (3)	149
N1—H1*N*⋯Cl1	0.86	2.57	3.003 (3)	112

Symmetry code: (i) 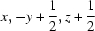.

**Fig. 1.**
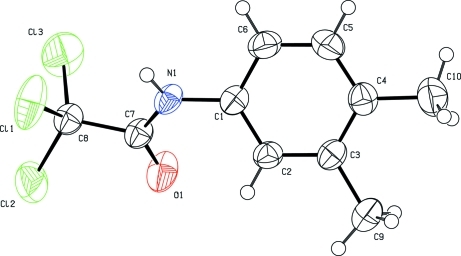
Molecular structure of the title compound, showing the atom labeling scheme and displacement ellipsoids drawn at the 50% probability level.

**Fig. 2.**
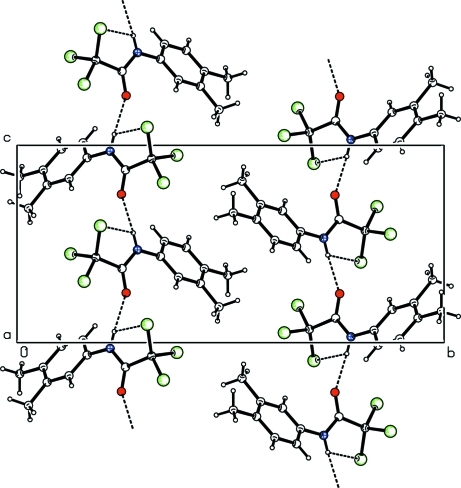
Molecular packing of the title compound with hydrogen bonding shown as dashed lines.

## supplementary crystallographic information

### Comment

As part of a study of the effect of ring and side chain substitutions on the crystal structures of aromatic amides (Gowda *et al.*, 2007; 2008; 2009), the structure of 2,2,2-trichloro-*N*-(3,4-dimethylphenyl)acetamide has been determined. The conformation of the N—H bond in the title compound is *anti* to the 3-methyl substituent in the aromatic ring similar to that observed with respect to the 3-chloro substituent in *N*- (3,4-dichlorophenyl)-2,2,2-trichloroacetamide (Gowda *et al.*, 2007), but in contrast to the *syn* conformation observed with respect to the 3-methyl substituent in *N*-(3,4-dimethylphenyl)acetamide (Gowda *et al.*, 2008). The conformation of the C=O bond in the structure is *anti* to the N—H bond similar to that observed in other amides. The amide H atom exhibits both N—H···Cl intramolecular and N—H···O intermolecular hydrogen bonding. The molecules in (I) are linked into infinite chains through intermolecular N—H···O hydrogen bonding (Table 1, Fig. 2).

### Experimental

The title compound  was prepared according to the literature method (Shilpa & Gowda, 2007). Single crystals were obtained from the slow evaporation of an ethanolic solution.

### Refinement

The H atoms were positioned with idealized geometry using a riding model with C—H = 0.93–0.96 Å, N—H = 0.86 Å, and were refined with isotropic displacement parameters  set to 1.2 times of the *U*_eq_ of the parent atom.

### Crystal data

**Table:**

C_10_H_10_Cl_3_NO	*F*(000) = 544
*M**_r_* = 266.54	*D*_x_ = 1.495 Mg m^−^^3^
Monoclinic, *P*2_1_/*c*	Mo *K*α radiation, λ = 0.71073 Å
Hall symbol: -P 2ybc	Cell parameters from 3672 reflections
*a* = 5.9003 (8) Å	θ = 2.3–27.6°
*b* = 20.843 (2) Å	µ = 0.75 mm^−^^1^
*c* = 9.996 (1) Å	*T* = 299 K
β = 105.53 (1)°	Prism, colourless
*V* = 1184.4 (2) Å^3^	0.46 × 0.40 × 0.30 mm
*Z* = 4	

### Data collection

**Table:**

Oxford Diffraction Xcalibur diffractometer with a Sapphire CCD detector	2407 independent reflections
Radiation source: fine-focus sealed tube	1952 reflections with *I* > 2σ(*I*)
graphite	*R*_int_ = 0.015
Rotation method data acquisition using ω and φ scans	θ_max_ = 26.4°, θ_min_ = 2.3°
Absorption correction: multi-scan (*CrysAlis RED*; Oxford Diffraction, 2007)	*h* = −7→7
*T*_min_ = 0.726, *T*_max_ = 0.807	*k* = −25→26
9395 measured reflections	*l* = −12→12

### Refinement

**Table:**

Refinement on *F*^2^	Primary atom site location: structure-invariant direct methods
Least-squares matrix: full	Secondary atom site location: difference Fourier map
*R*[*F*^2^ > 2σ(*F*^2^)] = 0.057	Hydrogen site location: inferred from neighbouring sites
*wR*(*F*^2^) = 0.151	H-atom parameters constrained
*S* = 1.08	*w* = 1/[σ^2^(*F*_o_^2^) + (0.0592*P*)^2^ + 1.4852*P*] where *P* = (*F*_o_^2^ + 2*F*_c_^2^)/3
2407 reflections	(Δ/σ)_max_ < 0.001
138 parameters	Δρ_max_ = 0.96 e Å^−^^3^
0 restraints	Δρ_min_ = −0.88 e Å^−^^3^

### Special details

**Table:**

Experimental. Absorption correction: CrysAlis RED (Oxford Diffraction, 2007) Empirical absorption correction using spherical harmonics, implemented in SCALE3 ABSPACK scaling algorithm.
Geometry. All e.s.d.'s (except the e.s.d. in the dihedral angle between two l.s. planes) are estimated using the full covariance matrix. The cell e.s.d.'s are taken into account individually in the estimation of e.s.d.'s in distances, angles and torsion angles; correlations between e.s.d.'s in cell parameters are only used when they are defined by crystal symmetry. An approximate (isotropic) treatment of cell e.s.d.'s is used for estimating e.s.d.'s involving l.s. planes.
Refinement. Refinement of *F*^2^ against ALL reflections. The weighted *R*-factor *wR* and goodness of fit *S* are based on *F*^2^, conventional *R*-factors *R* are based on *F*, with *F* set to zero for negative *F*^2^. The threshold expression of *F*^2^ > σ(*F*^2^) is used only for calculating *R*-factors(gt) *etc*. and is not relevant to the choice of reflections for refinement. *R*-factors based on *F*^2^ are statistically about twice as large as those based on *F*, and *R*- factors based on ALL data will be even larger.

### Fractional atomic coordinates and isotropic or equivalent isotropic displacement parameters (Å^2^)

**Table:**

	*x*	*y*	*z*	*U*_iso_*/*U*_eq_	
Cl1	1.2122 (2)	0.19521 (5)	0.59191 (11)	0.0833 (4)	
Cl2	1.1512 (2)	0.15721 (6)	0.31037 (12)	0.0939 (5)	
Cl3	0.8043 (2)	0.12567 (5)	0.45005 (16)	0.0919 (4)	
O1	0.8060 (5)	0.25550 (12)	0.2520 (2)	0.0643 (7)	
N1	0.8025 (4)	0.28196 (11)	0.4703 (2)	0.0403 (5)	
H1N	0.8600	0.2723	0.5563	0.048*	
C1	0.6475 (5)	0.33590 (13)	0.4375 (3)	0.0377 (6)	
C2	0.6550 (5)	0.37830 (13)	0.3319 (3)	0.0399 (6)	
H2	0.7657	0.3724	0.2819	0.048*	
C3	0.4996 (5)	0.42941 (13)	0.2998 (3)	0.0413 (6)	
C4	0.3341 (5)	0.43886 (14)	0.3749 (3)	0.0459 (7)	
C5	0.3325 (6)	0.39622 (17)	0.4814 (4)	0.0552 (8)	
H5	0.2243	0.4024	0.5329	0.066*	
C6	0.4852 (6)	0.34532 (15)	0.5131 (3)	0.0500 (7)	
H6	0.4796	0.3174	0.5848	0.060*	
C7	0.8629 (5)	0.24601 (13)	0.3758 (3)	0.0391 (6)	
C8	1.0067 (5)	0.18464 (14)	0.4322 (3)	0.0440 (7)	
C9	0.5074 (7)	0.47305 (17)	0.1813 (4)	0.0618 (9)	
H9A	0.6361	0.4609	0.1449	0.074*	
H9B	0.5285	0.5166	0.2138	0.074*	
H9C	0.3625	0.4695	0.1096	0.074*	
C10	0.1610 (7)	0.49358 (19)	0.3417 (4)	0.0662 (10)	
H10A	0.2428	0.5334	0.3672	0.079*	
H10B	0.0457	0.4884	0.3929	0.079*	
H10C	0.0843	0.4937	0.2441	0.079*	

### Atomic displacement parameters (Å^2^)

**Table:**

	*U*^11^	*U*^22^	*U*^33^	*U*^12^	*U*^13^	*U*^23^
Cl1	0.0948 (8)	0.0602 (6)	0.0657 (6)	0.0180 (5)	−0.0288 (5)	−0.0006 (4)
Cl2	0.1151 (9)	0.1008 (9)	0.0806 (7)	0.0593 (7)	0.0517 (7)	0.0208 (6)
Cl3	0.0866 (8)	0.0481 (5)	0.1423 (11)	−0.0182 (5)	0.0329 (7)	0.0060 (6)
O1	0.0981 (18)	0.0639 (14)	0.0272 (10)	0.0322 (13)	0.0104 (11)	−0.0009 (10)
N1	0.0564 (14)	0.0378 (12)	0.0244 (10)	0.0055 (10)	0.0067 (10)	0.0019 (9)
C1	0.0476 (15)	0.0331 (13)	0.0305 (13)	−0.0007 (11)	0.0074 (11)	−0.0030 (10)
C2	0.0472 (15)	0.0398 (14)	0.0344 (14)	0.0002 (12)	0.0141 (12)	0.0006 (11)
C3	0.0482 (16)	0.0346 (14)	0.0389 (14)	−0.0022 (12)	0.0078 (12)	0.0003 (11)
C4	0.0460 (16)	0.0386 (15)	0.0520 (17)	−0.0001 (12)	0.0113 (13)	−0.0036 (13)
C5	0.0551 (19)	0.0589 (19)	0.060 (2)	0.0054 (15)	0.0295 (16)	−0.0003 (16)
C6	0.0630 (19)	0.0491 (17)	0.0430 (16)	0.0002 (15)	0.0230 (14)	0.0058 (13)
C7	0.0475 (15)	0.0370 (14)	0.0305 (13)	0.0016 (12)	0.0064 (11)	−0.0009 (11)
C8	0.0501 (17)	0.0389 (15)	0.0418 (15)	0.0020 (12)	0.0100 (13)	0.0007 (12)
C9	0.073 (2)	0.0528 (19)	0.063 (2)	0.0143 (17)	0.0238 (18)	0.0199 (16)
C10	0.059 (2)	0.057 (2)	0.084 (3)	0.0139 (17)	0.0217 (19)	0.0043 (19)

### Geometric parameters (Å, °)

**Table:**

Cl1—C8	1.741 (3)	C4—C5	1.389 (5)
Cl2—C8	1.759 (3)	C4—C10	1.507 (4)
Cl3—C8	1.755 (3)	C5—C6	1.373 (5)
O1—C7	1.209 (3)	C5—H5	0.9300
N1—C7	1.327 (3)	C6—H6	0.9300
N1—C1	1.431 (3)	C7—C8	1.555 (4)
N1—H1N	0.8600	C9—H9A	0.9600
C1—C6	1.383 (4)	C9—H9B	0.9600
C1—C2	1.387 (4)	C9—H9C	0.9600
C2—C3	1.386 (4)	C10—H10A	0.9600
C2—H2	0.9300	C10—H10B	0.9600
C3—C4	1.395 (4)	C10—H10C	0.9600
C3—C9	1.504 (4)		
			
C7—N1—C1	123.9 (2)	O1—C7—N1	125.6 (3)
C7—N1—H1N	118.0	O1—C7—C8	118.7 (2)
C1—N1—H1N	118.0	N1—C7—C8	115.5 (2)
C6—C1—C2	119.6 (3)	C7—C8—Cl1	114.0 (2)
C6—C1—N1	118.7 (2)	C7—C8—Cl3	107.0 (2)
C2—C1—N1	121.7 (2)	Cl1—C8—Cl3	108.74 (17)
C3—C2—C1	120.8 (3)	C7—C8—Cl2	109.6 (2)
C3—C2—H2	119.6	Cl1—C8—Cl2	109.15 (17)
C1—C2—H2	119.6	Cl3—C8—Cl2	108.17 (17)
C2—C3—C4	120.0 (3)	C3—C9—H9A	109.5
C2—C3—C9	119.3 (3)	C3—C9—H9B	109.5
C4—C3—C9	120.8 (3)	H9A—C9—H9B	109.5
C5—C4—C3	118.1 (3)	C3—C9—H9C	109.5
C5—C4—C10	120.6 (3)	H9A—C9—H9C	109.5
C3—C4—C10	121.3 (3)	H9B—C9—H9C	109.5
C6—C5—C4	122.2 (3)	C4—C10—H10A	109.5
C6—C5—H5	118.9	C4—C10—H10B	109.5
C4—C5—H5	118.9	H10A—C10—H10B	109.5
C5—C6—C1	119.4 (3)	C4—C10—H10C	109.5
C5—C6—H6	120.3	H10A—C10—H10C	109.5
C1—C6—H6	120.3	H10B—C10—H10C	109.5
			
C7—N1—C1—C6	140.0 (3)	C4—C5—C6—C1	0.3 (5)
C7—N1—C1—C2	−39.4 (4)	C2—C1—C6—C5	0.6 (5)
C6—C1—C2—C3	−1.0 (4)	N1—C1—C6—C5	−178.9 (3)
N1—C1—C2—C3	178.5 (3)	C1—N1—C7—O1	3.8 (5)
C1—C2—C3—C4	0.4 (4)	C1—N1—C7—C8	−172.3 (2)
C1—C2—C3—C9	−177.9 (3)	O1—C7—C8—Cl1	145.1 (3)
C2—C3—C4—C5	0.5 (4)	N1—C7—C8—Cl1	−38.5 (3)
C9—C3—C4—C5	178.7 (3)	O1—C7—C8—Cl3	−94.6 (3)
C2—C3—C4—C10	−179.4 (3)	N1—C7—C8—Cl3	81.8 (3)
C9—C3—C4—C10	−1.1 (5)	O1—C7—C8—Cl2	22.4 (4)
C3—C4—C5—C6	−0.8 (5)	N1—C7—C8—Cl2	−161.1 (2)
C10—C4—C5—C6	179.0 (3)		

### Hydrogen-bond geometry (Å, °)

**Table:**

*D*—H···*A*	*D*—H	H···*A*	*D*···*A*	*D*—H···*A*
N1—H1N···O1^i^	0.86	2.14	2.917 (3)	149
N1—H1N···Cl1	0.86	2.57	3.003 (3)	112

Symmetry codes: (i) *x*, −*y*+1/2, *z*+1/2.
